# A new concept and a comprehensive evaluation of SYSMEX UF-1000i  flow cytometer to identify culture-negative urine specimens in patients with UTI

**DOI:** 10.1007/s10096-017-2964-1

**Published:** 2017-04-06

**Authors:** T. Monsen, P. Ryden

**Affiliations:** 1 Department of Clinical Microbiology, Umeå University Hospital, and University of Umeå, SE-90185 Umeå, Sweden; 2 Department of Mathematics and Mathematical Statistics, Umeå University, Umeå, Sweden; 3 Computational Life science Cluster (CLiC), Umeå University, Umeå, Sweden

**Keywords:** UTI, Screening, Urine culture, Flow-cytometry, Sysmex UF-1000i

## Abstract

Urinary tract infections (UTIs) are among the most common bacterial infections in men and urine culture is gold standard for diagnosis. Considering the high prevalence of culture-negative specimens, any method that identifies such specimens is of interest. The aim was to evaluate a new screening concept for flow cytometry analysis (FCA). The outcomes were evaluated against urine culture, uropathogen species and three conventional screening methods. A prospective, consecutive study examined 1,312 urine specimens, collected during January and February 2012. The specimens were analyzed using the Sysmex UF1000i FCA. Based on the FCA data culture negative specimens were identified in a new model by use of linear discriminant analysis (FCA-LDA). In total 1,312 patients were included. In- and outpatients represented 19.6% and 79.4%, respectively; 68.3% of the specimens originated from women. Of the 610 culture-positive specimens, *Escherichia coli* represented 64%, enterococci 8% and *Klebsiella spp*. 7%. Screening with FCA-LDA at 95% sensitivity identified 42% (552/1312) as culture negative specimens when UTI was defined according to European guidelines. The proposed screening method was either superior or similar in comparison to the three conventional screening methods. In conclusion, the proposed/suggested and new FCA-LDA screening method was superior or similar to three conventional screening methods. We recommend the proposed screening method to be used in clinic to exclude culture negative specimens, to reduce workload, costs and the turnaround time. In addition, the FCA data may add information that enhance handling and support diagnosis of patients with suspected UTI pending urine culture.

## Introduction

Urinary tract infections (UTIs) are among the most common infections in community and hospitalized patients with more than 175 million UTI incidences worldwide [1]. UTI is caused by pathogenic microorganisms which induce infection and an inflammatory response with presence of leukocyturia and erythrocyturia. However, UTIs are often harmless and self-eradicated [2]. Nevertheless, UTIs are associated with high morbidity and costs and especially among patients with diabetes [3]. In the United States UTIs account annually for more than 7 million physician visits, more than 1 million emergency department visits and more than 100,000 hospitalizations. The estimated annual cost for treatment is calculated to more than US $1 billion and the indirect cost estimated to approximately $1.6 billion [4, 5]. Furthermore, 15% of all antibiotics prescribed to outpatients and data from some European countries also suggest a similar rate [6, 7].

In a large German study on patients with diabetes mellitus type 2 the cost was estimated to €316 per UTI event with a total increased cost of €3916 per patient in the UTI group compared with the non UTI group [3].

For diagnosis of UTI symptoms such as urgency, dysuria, frequent urination, back pain, leukocyturia and a positive nitrite test are considered reliable indicators [2, 8]. However, urine culture is the gold standard for diagnosis of UTI but is laborious and moderately costly, with a turnaround time of 24 to 48 h [9].

In clinical laboratories, urine specimens are among the most commonly encountered and approximately a quarter to more than half are considered culture negative [9, 10]. For this reason, any screening method that identifies and excludes urine specimens with non-significant bacteriuria would be of great interest [11–13].

Flow cytometry analysis (FCA) is a promising method to identify and enumerate bacteria, leukocytes, erythrocytes and other particles in urine. The second generation automated FCA instrument, the Sysmex UF-1000i (Medical Electronics, Kobe, Japan) has a separate detection channel for bacteria with improved sensitivity (SE) and specificity (SP) [11–15].

Using FCA, we have recently evaluated the inflammatory response of leukocytes and erythrocytes in urine for different pathogens in patients with suspected UTI [9]. Based on the dataset we now aimed to set up and assess a new screening model to identify and rule out culture negative urine specimens in patients with suspected UTI prior to culture. The prerequisites for the screening model are high SE to prevent specimens with significant bacteriuria (SBU) from being erroneously classified as negative (i.e. false negatives) and a relatively high SP to prevent unnecessary culturing. The present screening model was evaluated against three conventional screening methods [11, 16, 17].

## Material and methods

### Collection of urine specimens

A prospective consecutive multicenter study was conducted during January and February 2012 which analyzed urine specimens from in- and outpatients. The specimens were collected in non-preservative tubes, stored and transported at ≤6 °C to the Department of Clinical Microbiology at the University Hospital of Umeå for analysis. All specimens were from the county of Västerbotten, Sweden.

### Urinalysis

All specimens underwent FCA and urine culture within 3 h of arrival to the laboratory. Specimens that arrived after 4 PM were analyzed with flow cell analysis (FCA) and stored at +6 °C until cultured the following morning. Excluded were specimens from pregnant women, urinary catheter and those that lacked complete FCA data.

Flow cytometry analysis was performed using the UF-1000i instrument (Sysmex, TOA Medical Electronics, Kobe, Japan), supported by the Sysmex software version of 00–15 [9]. The screening model was built on the observed bacterial and leukocyte counts.

### Urine culture

Gram-negative and Gram-positive uropathogens were identified by Brilliance™ UTI agar (Oxoid Ltd., Basingstoke, UK) biochemical tests, and urine culture was performed as previously described [9].

### Species identification

Isolates were identified in specimens with presence of ≥10^6^ colony forming units/L (CFU/L) and those with mixed flora (with both gram negative and gram positive bacteria) with a dominating pathogen (i.e. bacterial count at least 10 times higher than any other species) [9].

### Significant bacteriuria

SBU was defined in accordance with European guidelines (at ≥10^6^ CFU/L of an uropathogen with acute uncomplicated cystitis) [7] and was the gold standard in the present article [18, 19]. In patients with <10^8^ (≥10^6^ and 10^7^ CFU/L) and where the presence or absence of UTI symptoms could not be determined, the specimens were classified indeterminant.

Evaluations were also done when SBU was defined as ≥10^7^ or ≥10^8^ CFU/L of a uropathogen, respectively, irrespective of presence of UTI symptoms.

### Ethical approval

All procedures were in accordance with institutional and national ethical standards and the Helsinki declaration.

### Statistical analysis

Urine specimens analyzed with FCA and urine culture were used to derive a decision rule that based on FCA data determines which specimens should be cultured. Screening methods based on bacterial counts (BC) and white blood cell counts (WBC) have been suggested by Jolkkonen, Manoni and De Rosa [11, 16, 17]. Here, specimens are cultured if either BC > *a* or WBC > *b*, where *a* and *b* are the method specific cut-off values: Jolkonnen: *a* = 405, *b* = 16; Manoni: *a* = 125, *b* = 40; De Rosa: *a* = 170, *b* = 150.

**Fig. 1:**
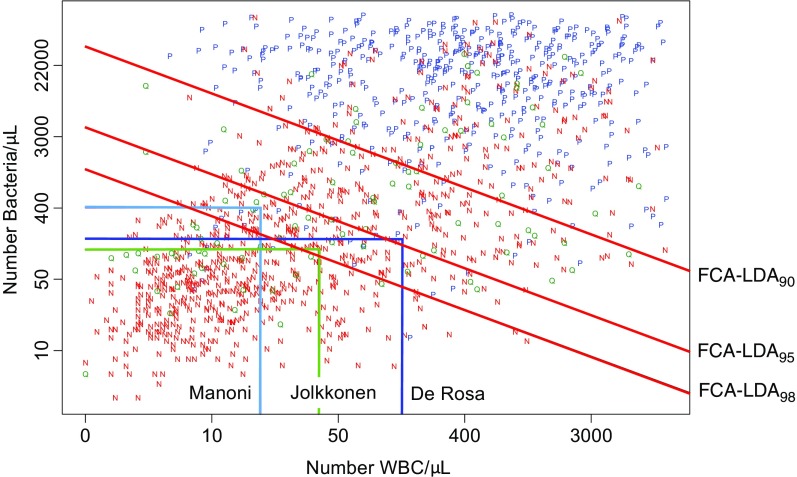
Plot of 1,312 urine specimens with significant, non-significant and indeterminant bacteriuria and the classification criteria or the different screening methods examined. Specimens above the classification line were predicted as positives and send to culture. P (*blue color*) = “positive specimens", i.e. specimens with significant bacteriuria when urinary tract infection was defined according to European guidelines. N (*red colour*) = “negative specimens", i.e. specimens with non-significant bacteriuria when urinary tract infection was defined according to European guidelines. Q (*green colour*) = “indeterminant/questionable” specimens, i.e. specimens with indeterminant significant bacteriuria when urinary tract infection was defined according to European guidelines (mainly specimens that lacked information regarding UTI symptoms), number of bacteria (Bact = Y-axis) and white blood cells (WBC = X- axis) estimated by flow cell instrument per uL. *Red lines* represent the cut off for linear discriminant analysis at: 98% sensitivity (FCA-LDA_98_), 95% sensitivity (FCA-LDA_95_) and 90% sensitivity (FCA-LDA_90_). *Dark blue color* represents the cut-off values according to De Rosa et al. (cut off: bacteria 170, white blood cells 150). *Green color* represents the cut-off values according to Manoni et al. (cut off: bacteria 125, white blood cells 40). *Light blue color* represents the cut-off values according to Jolkkonen et al. (cut off: bacteria 405, white blood cells 16)

We suggest the FCA-LDA approach that uses linear discriminant analysis (LDA) on the FCA variables BC and WBC [20]. The FCA-LDA rule was derived using data from specimens that were concluded to be significant bacteriuria or non-significant bacteriuria and with BC < 5000. A LDA-model was fitted to the log-transformed FCA-values, assuming homoscedasticity and proportional priors, which resulted in a decision rule: [ln (BC) > α + βln(WBC)]. The intercept *α* was tuned so that the rule had the desired sensitivity (SE). The decision rule is a line, where specimens above the line are cultured (Fig. 1).

The methods were evaluated in terms of their SE and specificity (SP), negative and positive predictive values (PPV and NPV), the number of cultured specimens and the relative cost (RC) compared to the standard procedure. RC was calculated under the assumption that the running cost of culture is five times as high as running FCA. The evaluations were performed for different definitions of UTI.

In a similar style as described above a LDA-model, based on BC and the variable B-FSC (the forward scatter / “particle size”), was derived to determine if the specimens’ bacteria were gram positive or gram negative. This resulted in a classification rule: [ln (BC) > α + β ln(B-FSC)] where specimens were predicted gram positive if the inequality was valid and gram-negative otherwise.

## Results

In total, 1587 urine specimens were eligible for FCA and urine culture. Two hundred seventy-five specimens (17.3%) were excluded: 125 urinary catheters, 128 specimens from pregnant women, 18 that lacked clinical information and four specimens with candidauria. In total, 1312 specimens were enrolled in the present study (Fig. 1).

Among genders, 68.3% (*p* < 0.001) of the specimens originated from women with a mean age of 58.9 years (standard deviation 25.5) versus 31.7% and 61.7 years (20.8) for men. Outpatients represented 79.4% and inpatients 20.6%. The demographic information of the dataset is presented in Table 1.

**Table 1:** Demographic data of 1,312 patients and urine specimens enrolled in the study

Characteristic	Total^a^	Women^a^	Men^a^
Age (mean)	58.9	57.6	61.7
Outpatients (%)	79.4	82.4	72.3
Specimens included, *n* (%)	1312	896 (68)	416 (32)
Growth distribution at culture			
> 10^8^ colony forming units per liter	447 (34)	359 (80)	88 (20)
10^7−^10^8^ colony forming units per liter	370 (28)	271 (73)	99 (27)
10^6−^10^7^ colony forming units per liter	288 (22)	193 (67)	95 (33)
No growth	207 (16)	73 (35)	134 (65)
SBU^b^ , *n* (%)	473	380 (80)	93 (20)
Gram-negative	405 (86)	337 (83)	68 (17)
*Escherichia coli*	337 (71)	288 (85)	49 (15)
*Klebsiella* spp.	29 (6)	24 (83)	5 (17)
Other	39 (8)	25 (64)	14 (36)
Gram-positive	68 (14)	43 (63)	26 (37)
*Staphylococcus* spp.	24 (5)	17 (71)	7 (29)
*Enterococcus* spp.	32 (7)	18 (56)	14 (44)
Other	12 (2)	8 (67)	4 (33)
Indeterminant SBU^b^, *n* (%)	98	68 (69)	30 (31)
Gram-negative	74 (75)	50 (69)	24 (31)
*Escherichia coli*	54 (55)	37 (69)	17 (31)
*Klebsiella* spp.	13 (13)	9 (69)	4 (31)
Other	7 (7)	4 (57)	3 (43)
Gram-positive	24 (25)	18 (76)	6 (24)
*Staphylococcus* spp.	2 (2)	1 (50)	1 (50)
*Enterococcus* spp.	15 (15)	10 (67)	5 (33)
Other	7 (7)	7 (100)	0 (0)
Non significant SBU^b^, *n* (%)	741	448 (60)	293 (40)
Gram-negative	10 (1)	7 (70)	3 (30)
*Escherichia coli*	2 (0)	1 (50)	1 (50)
*Klebsiella* spp.	3 (0)	2 (66)	1 (33)
Other	5 (1)	4 (80)	1 (20)
Gram-positive	29 (4)	24 (83)	5 (17)
*Staphylococcus* spp.	5 (1)	2 (40)	3 (60)
*Enterococcus* spp.	4 (1)	4 (100)	0 (0)
Other	20 (3)	18 (90)	2 (10)
Mixed flora Gram-negative	202 (27)	148 (73)	54 (27)
Mixed flora Gram-positive	293 (39)	196 (67)	97 (33)
No growth	207 (28)	73 (35)	134 (65)

^a^The percentage (%) represents the figures within each sub-group. The columns *Women* and *Men* represent the relative distribution between the genders

^b^Significant bacteriuria according to European guidelines (SBU), indeterminant according to European guidelines and non-significant bacteriuria according to European guidelines

When UTI was defined according to European guidelines, 36.0% (473/1312 specimens) had significant bacteriuria, 56.5% (741/1312 specimens) had non significant bacteriuria and 7.5% (98/1312 specimens) were classified as indeterminant, due to lack of clinical information.

*E. coli* was the most predominant uropathogen, representing 71.2% (337/473) of the uropathogens followed by *Enterococcus faecalis* 6.8%, *Klebsiella pneumoniae* 6.1% and coagulase-negative staphylococci 5.1% (Tables 1 and 2).

**Table 2:** Outcome of the four screening methods of 1,312 urine specimens examined with flow cytometry analysis when UTI was defined according to European guidelines

Group^a^	Bacteria	SBU			Methods evaluated^b^																	
					Jolkkonen			Manoni			De Rosa			FCA-LDA_98_			FCA-LDA_95_			FCA-LDA_90_		
		Pos^c^	Neg	Q	SE	SP	NC	SE	SP	NC	SE	SP	NC	SE	SP	NC	SE	SP	NC	SE	SP	NC
EC	*Escherichia coli*	337	2	54	**99**	**0**	**16**	99	0	15	**98**	**0**	**23**	99	0	20	**97**	**0**	**38**	93	100	70
KC	*Acinetobacter spp*.	0	1	0		**0**	**0**		0	0		**100**	**1**		0	0		**100**	**1**		100	1
KC	*Citrobacter spp*.	9	0	0	**100**		**0**	100		0	**100**		**0**	100		0	**100**		**0**	89		1
KC	*Citrobacter freundii*	1	0	0	**100**		**0**	100		0	**100**		**0**	100		0	**100**		**0**	100		0
KC	*Enterobacter spp*.	2	1	2	**100**	**0**	**1**	100	0	1	**100**	**0**	**1**	100	0	1	**100**	**0**	**1**	100	0	1
KC	*Other Klebsiella spp*.	1	0	1	**100**		**1**	100		1	**100**		**1**	100		1	**100**		**1**	100		1
KC	*Klebsiella oxytoca*	8	1	1	**100**	**0**	**0**	100	0	0	**100**	**0**	**0**	100	0	0	**100**	**0**	**0**	100	0	1
KC	*Klebsiella pneumoniae*	20	2	11	**95**	**100**	**8**	100	50	6	**100**	**50**	**7**	95	100	8	**95**	**100**	**9**	95	100	9
KC	*Pantoea spp*.	1	0	0	**100**		**0**	100		0	**100**		**0**	100		0	**100**		**0**	100		0
KC	Aggregated	42	5	15	**98**	**40**	**10**	100	20	8	**100**	**40**	**10**	98	40	10	**98**	**60**	**12**	95	60	14
PR	*Morganella morganii*	2	1	2	**100**	**100**	**1**	100	100	1	**100**	**100**	**1**	100	100	1	**100**	**100**	**2**	100	100	3
PR	*Proteus mirabilis*	9	1	2	**100**	**0**	**0**	100	0	0	**100**	**100**	**1**	89	0	1	**89**	**100**	**2**	89	100	3
PR	*Proteus vulgaris*	2	1	0	**100**	**0**	**0**	100	0	0	**100**	**0**	**0**	100	0	0	**100**	**0**	**0**	100	0	0
PR	*Providencia rettgeri*	1	0	0	**100**		**0**	100		0	**100**		**0**	100		0	**100**		**0**	100		0
PR	Aggregated	14	3	4	**100**	**33**	**1**	100	33	1	**100**	**67**	**2**	93	33	2	**93**	**67**	**4**	93	67	6
PS	*Pseudomonas aeruginosa*	11	0	0	**91**		**1**	100		0	**100**		**0**	91		1	**91**		**1**	91		1
PS	Other *Pseudomonas spp*.	0	0	1			**0**			0			**0**			0			**0**			0
PS	Aggregated	11	0	1	**91**		**1**	100		0	**100**		**0**	91		1	**91**		**1**	91		1
ST	CoN*S*	13	4	0	**100**	**0**	**0**	100	25	1	**100**	**25**	**1**	100	25	1	**100**	**25**	**1**	85	25	3
ST	*Staphylococcus aureus*	8	1	1	**88**	**0**	**1**	100	0	0	**100**	**0**	**0**	100	0	0	**75**	**0**	**2**	63	100	5
ST	*Staphylococcus saprophyticus*	3	0	1	**100**		**0**	100		0	**100**		**0**	100		0	**100**		**0**	67		1
ST	Aggregated	24	5	2	**96**	**0**	**1**	100	20	1	**100**	**20**	**1**	100	20	1	**92**	**20**	**3**	75	40	9
EN	*Enterococcus faecalis*	28	4	10	**93**	**0**	**6**	96	0	4	**93**	**0**	**6**	96	0	5	**82**	**25**	**11**	71	75	17
EN	*Enterococcus faecium*	4	0	5	**100**		**2**	100		1	**100**		**2**	100		2	**100**		**3**	75		6
EN	Aggregated	32	4	15	**94**	**0**	**8**	97	0	5	**94**	**0**	**8**	97	0	7	**84**	**25**	**14**	72	75	23
SR	Alpha-hemolytic streptococci	3	6	0	**67**	**17**	**2**	67	17	2	**67**	**17**	**2**	67	17	2	**67**	**17**	**2**	67	33	3
SR	*Gemella haemolysans*	1	0	0	**100**		**0**	100		0	**100**		**0**	100		0	**100**		**0**	100		0
SR	*Streptococcus* Group A	0	0	1			**0**			0			**0**			0			**0**			1
SR	*Streptococcus* Group G	0	0	3			**0**			0			**0**			0			**1**			2
SR	*Streptococcus* Group C	1	0	3	**100**		**0**	100		0	**100**		**0**	100		0	**100**		**0**	0		2
SR	Aggregated	5	6	7	**80**	**17**	**2**	80	17	2	**80**	**17**	**2**	80	17	2	**80**	**17**	**3**	60	33	8
GB	*Streptococcus agalactiae*	6	14	0	**83**	**36**	**6**	100	29	4	**100**	**36**	**5**	100	50	7	**67**	**64**	**11**	50	79	14
OT	Diphtheroid rod	1	0	0	**100**		**0**	100		0	**100**		**0**	100		0	**100**		**0**	100		0
OT	*Haemophilus influenzae*	1	0	0	**100**		**0**	100		0	**100**		**0**	100		0	**100**		**0**	100		0
OT	Aggregated	2	0	0	**100**		**0**	100		0	**100**		**0**	100		0	**100**		**0**	100		0
NE	Culture negative	0	207	0		**64**	**132**		64	132		**77**	**160**		80	165		**87**	**181**		96	198
MP	Mixed flora G+	0	293	0		**34**	**100**		37	107		**43**	**127**		45	133		**57**	**166**		78	229
MN	Mixed flora G-	0	202	0		**31**	**62**		27	55		**41**	**82**		36	73		**59**	**119**		79	160
All	Total	473	741	98	**98**	**41**	**339**	99	41	330	**98**	**51**	**420**	98	52	421	**95**	**65**	**552**	90	83	732

*SBU* significant bacteriuria, *SE* sensitivity, *SP* specificity, *NC* not cultured, i.e. specimens screened as culture negative, *spp* species, *CoNS* coagulase negative staphylococci

^a^Group = bacteria or bacterial groups. EC = E. coli; KC = *Klebsiella/Citrobacter* group; PR = *Proteus* group; PS = *Pseudomonas* group; ST = Staphylococcal group; SR = Streptococci group; GB = *Streptococcus agalactiae* (GBS); OT = other bacteria; NE = culture negative; MP = mixed Gram-positive flora; MN = mixed Gram-negative flora; All = total

^b^Methods evaluated: Jolkkonen et al., Manoni et al., De Rosa et al., linear discriminant analysis at 98, 95 and 90% sensitivity, respectively (FCA-LDA_98,_ FCA-LDA_95,_ FCA-LDA_90_)

^c^Pos = SBU when urinary tract infection was defined according to European guidelines; Neg = non SBU according to European guidelines; Q = indeterminant (questionable) significant bacteriuria according to European guidelines

FCA-LDA decision rules, using BC and WBC as the predictors and controlling the SE at 90%, 95% and 98%, respectively, were derived, i.e.

- FCA-LDA_98_: ln(BC) > 10.46–0.65 ln (WBC)
- FCA-LDA_95_: ln(BC) > 8.22–0.65 ln (WBC)
- FCA-LDA_90_: ln(BC) > 7.07–0.65 ln (WBC)

Recall that a specimen is cultured if the inequality is fulfilled. The FCA-LDA-rules were compared to the bivariate decision rules suggested by Jolkkonen, Manoni and De Rosa [11, 16, 17]. The six decision rules and their outcome are shown in Fig. 1.

### Evaluation when SBU was defined according to European guidelines

**Fig. 2:**
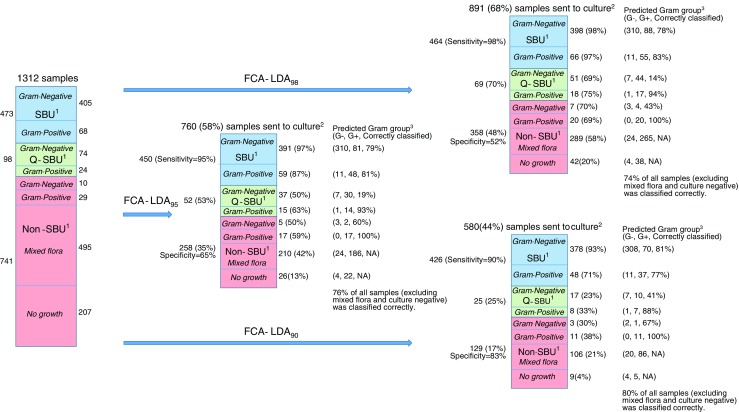
Outcome of the 1312 urine specimens examined by flow cytometry analysis and samples sent to culture after screening using linear discriminant  analysis at 90, 95 and 98% sensitivity when significant bacteriuria was defined according to European guidelines. *Blue arrows*: represent the outcome of linear discriminant analysis at 98, 95 and 90% sensitivity, respectively (FCA-LDA_98,_ FCA-LDA_95,_ FCA-LDA_90_) when urinary tract infection was defined according to European guidelines. G+ = gram positive species. G– = gram negative species. ^1^SBU = significant bacteriuria,* blue-colored boxes*; Q-SBU = indeterminant (questionable) SBU, *green-colored boxes*; Non SBU, *red-colored boxes*. ^2^Number of specimens send to culture when FCA-LDA selection was applied. ^3^ Specimens send to culture was classified as Gram negative or Gram positive bacteria

Overall, when the FCA-LDA_98_ decision rule was used and UTI was defined according to European guidelines (at ≥10^6^ CFU/L of an uropathogen with acute uncomplicated cystitis) [7] then the screen resulted in 98% SE (464 true positive specimens), 52% SP (385 true negative specimens) and 32% (421 specimens) were identified as culture negative (Fig. 2, total data). The corresponding numbers for the more restrictive inclusion rules FCA-LDA_95_ and FCA-LDA_90_ were (95%, 65%, and 42%) and (90%, 83%, and 56%), respectively (Fig. 2, Table 2). In comparison, the screening methods by Jolkkonen, Manoni and De Rosa had similar SE as for FCA-LDA_98_; however, the Jolkkonen’s and Manoni’s methods had lower SP (41%) compared to FCA-LDA_98_ (52%) and De Rosa (51%). The outcome of the six decision rules are presented in Table 2. In general, the screening methods had relative high SE for *E. coli* (0–3% above average SE for the examined methods) and *Klebsiella/Citrobacter* groups (KC-group 0–5% above average SE). Relative low SE was found for the enterococci group (EN group −1 to −18% below average SE) and the streptococci group (SR group −18 to −30% below average SE for the examined methods ; Table 2).

Screening at SBU defined according to European guidelines yielded higher SE and SP than UTI defined at ≥10^7^ and ≥10^8^ /CFU/L (Table 3).

**Table 3:** Outcome of the four screening methods to rule out culture negative urine specimens among patients with suspected UTI

SBU^a^	Measures (%)	Methods evaluated^b^					
		Jolkkonen	Manoni	De Rosa	FCA-LDA_98_	FCA-LDA_95_	FCA-LDA_90_
European Guidelines	SE	98	99	98	98	95	90
	SP	41	41	51	52	65	83
	PPV	51	52	56	56	63	77
	NPV	96	98	98	98	95	93
	NC^c^	26	25	32	32	42	56
	RC^d^	94	95	88	88	78	64
≥10^7^ CFU/L	SE	94	96	94	94	90	81
	SP	42	42	53	52	67	84
	PPV	56	56	61	61	68	80
	NPV	91	93	92	92	89	85
≥10^8^ CFU/L	SE	99	100	100	99	98	95
	SP	38	38	48	48	62	81
	PPV	44	44	49	49	56	71
	NPV	99	100	100	99	99	97

*SE* sensitivity, *SP* specificity, *PPV* positive predictive value, *NPV* negative predictive value

^a^SBU = Definition of significant bacteriuria according to European guidelines, ≥10^7^ and ≥10^8^ colony forming units per liter in urine at culture

^b^Comparing screening methods according to Jolkkonen et al., Manoni et al., De Rosa et al. Flow cell analysis-linear discriminant analysis at 98% sensitivity (FCA-LDA_98_), FCA-LDA_95_ = at 95% sensitivity, and FCA-LDA_90_ = at 90% sensitivity

^c^NC = not cultured (%): specimens identified as culture negative by the screening method

^d^RC = relative cost of the screening method (%) compared to the gold standard procedure (100%) when all specimens are cultured. A calculated cost of, e.g. 78%, represents a 22% cost reduction when FCA screening was included in its cost

At FCA-LDA_98_, FCA- LDA_95_ and FCA- LDA_90_ screening 9, 23 (9 + 14) and 47 (23 + 24) specimens proved false negative, respectively, representing 0.7, 1.7 and 3.6% of the specimens. Information related to the false negative specimens is presented in Table 4. For the FCA-LDA_95_ screen, seven of the false negative specimens had 10^8^ CFU/L, eight 10^6^ and 10^8^ CFU/L each. Patients 2, 3, 7, 9, 10, 16 had all low WBC- and elevated bacterial counts indicating asymptomatic bacteriuria in these patients. Patient 8 had “high” WBC and red blood cell (RBC) counts but low bacterial counts despite presence of *Proteus mirabilis* at 10^8^ CFU/L at culture. Of the 47 false negative specimens, 72% (34/47) originated from women.

**Table 4:** Data associated with the 47 false negative urine specimens present in linear discriminatory analysis at 98%, 95% and 90% sensitivity

Sensitivity (%)	Patient	Gender	Patient age (years)	Unit	Specimen	10^n^ CFU/L^a^	Bacteria^b^	Nitrit-test^c^	UTI^d^ Symptom	Comments / clinical information	RBC^e^	WBC^f^	Bacterial counts^g^
0.98	1	M	32	HCC	IC	7	α-hemolytic str^m^			IC	2	5	49
0.98	2	F	6	HCC	MSU	7	*Escherichia coli*		P	BSU	5	6	127
0.98	3	M	75	Hosp.	IC	6	*Escherichia coli*				10	4	101
0.98	4	M	73	HCC	MSU	6	*Escherichia coli*		P	Increased CRP, BSU	4	6	69
0.98	5	F	80	HCC	MSU	6	*Escherichia coli*			500 WBC^h^	22	18	122
0.98	6	M	91	HCC	MSU	6	*Ent. faecalis*		P	Urgency	4	5	38
0.98	7	F	78	HCC	MSU	8	*Klebsiella pneum*.				1	7	224
0.98	8	F	23	Hosp.	MSU	8	*Proteus mirabilis*			Smelly urine, increased WBC^h^	65	170	9
0.98	9	F	29	HCC	MSU	8^M7^	*Ps. aeruginosa*			Ante-natal clinic	36	8	172
0.95	10	F	54	HCC	MSU	7	*Escherichia coli*				37	11	343
0.95	11	M	60	HCC	MSU	7	*Escherichia coli*		P	Urgency	74	69	76
0.95	12	F	5	HCC	MSU	6	*Escherichia coli*		P	Cystit symptom	18	423	62
0.95	13	F	36	HCC	MSU	6	*Escherichia coli*	+			4	81	131
0.95	14	F	68	HCC	MSU	6	*Escherichia coli*		P	Urgency, 1 h BID	20	244	33
0.95	15	F	75	HCC	MSU	6	*Escherichia coli*		P	Urgency	7	334	40
0.95	16	M	69	Hosp.	MSU	8	*Ent. faecalis*				8	8	290
0.95	17	M	79	HCC	MSU	8^M6^	*Ent. faecalis*				110	98	179
0.95	18	F	89	Hosp.	MSU	7^M6^	*Ent. faecalis*		P	Urgency	12	65	152
0.95	19	F	6	HCC	MSU	7^M6^	*Ent. faecalis*		P	Frequency	5	20	357
0.95	20	F	79	Hosp.	MSU	8	*Str. agalactiae*		P	BSU, urgency	18	14	364
0.95	21	F	50	HCC	MSU	8	*Str. agalactiae*				10	32	220
0.95	22	F	89	Hosp.	MSU	7	*S. aureus*			100 WBC^h^	6	11	236
0.95	23	F	37	HCC	MSU	7	*S. aureus*		P	Urgency	7	29	414
0.90	24	F	29	HCC	MSU	6	*Citrobacter spp*.				4	144	348
0.90	25	M	70	Hosp.	IC	7	CoNS			Fever	108	79	1085
0.90	26	M	79	HCC	IC	7	CoNS			UTI symptom. Neurogenic urine bladder disease	70	2495	82
0.90	27	M	20	HCC	MSU	8^M7^	*Escherichia coli*		P	BSU	4	210	467
0.90	28	F	26	HCC	MSU	8	*Escherichia coli*			500 WBC^h^	4	190	234
0.90	29	M	30	HCC	MSU	8	*Escherichia coli*				1	89	471
0.90	30	F	36	HCC	MSU	8	*Escherichia coli*	−	P	Urgency,	26	55	1334
0.90	31	F	47	HCC	MSU	8	*Escherichia coli*		P	Urgency, BSU	2	10	7478
0.90	32	F	47	HCC	MSU	8	*Escherichia coli*				87	28	2122
0.90	33	F	74	HCC	MSU	8^M6^	*Escherichia coli*	+			5	20	3684
0.90	34	F	92	HCC	MSU	8	*Escherichia coli*			UTI?	26	122	1382
0.90	35	F	35	HCC	MSU	7^M6^	*Escherichia coli*			500WBC^h^, 25RBC^h^	43	893	101
0.90	36	F	48	HCC	MSU	7	*Escherichia coli*			500 WBC^h^, 50 RBC^h^	4	288	785
0.90	37	F	70	HCC	MSU	7	*Escherichia coli*	+			8	139	186
0.90	38	F	33	HCC	MSU	6	*Escherichia coli*		P	500 WBC^h^, 200 RBC^h^	14	1113	151
0.90	39	F	71	HCC	MSU	6	*Escherichia coli*		P	BID 4 h, BSU, Urgency	6	52	284
0.90	40	M	62	HCC	MSU	8	*Ent. faecalis*			Urgency	2	6	1913
0.90	41	M	72	HCC	MSU	8	*Ent. faecalis*			Fever	17	25	981
0.90	42	F	73	HCC	MSU	8^M7^	*Ent. faecalis*		P	Urgency	12	35	2449
0.90	43	F	56	Hosp.	MSU	8^M6^	*Ent. faecium*			Neutropenia	78	12	1116
0.90	44	F	82	HCC	MSU	8^M6^	*Str. agalactiae*		P	Urgency	42	49	2339
0.90	45	F	70	Hosp.	IC	8	*S. aureus*			Infection?	40	6	2440
0.90	46	F	26	HCC	MSU	8^M6^	*S. saprophyticus*		P		9	155	1119
0.90	47	F	60	HCC	MSU	8	Streptococci				14	273	410

*M* male, *F* female, *HCC* heath care centre, *Hosp* hospital, *MSU* mid stream urine, *IC* intermittent catheterized, *α-hemolytic str* alpha haemolytic streptococci, *BSU* burning sensation during urination, *CoNS* coagulase negative staphylococci

^a^M6, M7 represents mixed flora at 10^6^, 10^7^ / colony forming units/L, respectively 10^n^ the numbers 6, 7, 8 represents 10^6^, 10^7^ and 10^8^ colony forming units/L, respective of bacteria quantified at urine culture. M = mixed flora, i.e. 8^M7^ = specimens with 10^8^ colony forming units/L of the dominating uropathogen and mixed flora 10^6^ colony forming units/L.

^b^Bacteria/uropathogen identified at culture

^c^ + = positive test, − = negative test

^d^UTI (urinary tract symptoms): + P = present

^e^RBC = red blood cells counted in flow cytometry analysis × 10^6^/L

^f^WBC = white blood cells counted in flow cytometry analysis × 10^6^/L

^g^Bacterial counts estimated in the flow cytometry analysis ×10^6^/L^h^WBC = white blood cells, RBC = red blood cells estimated at clinic/health care centre

### Evaluation when SBU was defined at ≥10^7^ and ≥10^8^ CFU/L

Overall, defining UTI at ≥10^7^ CFU/L of an uropathogen, irrespective of presence of UTI symptoms, resulted in lower sensitivities (3–9%) and similar specificities in comparison when SBU was defined according to European guidelines (Tables 3 and 5). Defining UTI as ≥10^8^ CFU/L (irrespective of presence of UTI symptoms) resulted in higher sensitivities (1–5%) and lower specificities (2–4%) in comparison to European guidelines (Tables 3 and 6).

**Table 5:** Outcome of the four screening methods of 1,312 urine specimens examined with flow cytometry analysis when UTI was defined as growth of ≥10^7^ colony forming units per liter at culture

Group^a^	Bacteria	SBU		Methods evaluated^b^																	
				Jolkkonen			Manoni			De Rosa			FCA-LDA_98_			FCA-LDA_95_			FCA-LDA_90_		
		Pos^c^	Neg	SE	SP	NC	SE	SP	NC	SE	SP	NC	SE	SP	NC	SE	SP	NC	SE	SP	NC
EC	*Escherichia coli*	374	19	**97**	**26**	**16**	98	32	15	**96**	**47**	**23**	97	37	20	**94**	**84**	**39**	86	100	70
KC	*Acinetobacter spp*.	0	1		**0**	**0**		0	0		**100**	**1**		0	0		**100**	**1**		100	1
KC	*Citrobacter spp*.	8	1	**100**	**0**	**0**	100	0	0	**100**	**0**	**0**	100	0	0	**100**	**0**	**0**	100	100	1
KC	*Citrobacter freundii*	1	0	**100**		**0**	100		0	**100**		**0**	100		0	**100**		**0**	100		0
KC	*Enterobacter spp*.	4	1	**100**	**100**	**1**	100	100	1	**100**	**100**	**1**	100	100	1	**100**	**100**	**1**	100	100	1
KC	*Other Klebsiella*	2	0	**50**		**1**	50		1	**50**		**1**	50		1	**50**		**1**	50		1
KC	*Klebsiella oxytoca*	10	0	**100**		**0**	100		0	**100**		**0**	100		0	**100**		**0**	90		1
KC	*Klebsiella pneumoniae*	29	4	**86**	**100**	**8**	90	75	6	**86**	**75**	**7**	86	100	8	**83**	**100**	**9**	83	100	9
KC	*Pantoea spp*.	1	0	**100**		**0**	100		0	**100**		**0**	100		0	**100**		**0**	100		0
KC	Aggregated	55	7	**91**	**71**	**10**	93	57	8	**91**	**71**	**10**	91	71	10	**89**	**86**	**12**	87	100	14
PR	*Morganella morganii*	4	1	**100**	**100**	**1**	100	100	1	**100**	**100**	**1**	100	100	1	**75**	**100**	**2**	50	100	3
PR	*Proteus mirabilis*	11	1	**100**	**0**	**0**	100	0	0	**100**	**100**	**1**	91	0	1	**91**	**100**	**2**	82	100	3
PR	*Proteus vulgaris*	3	0	**100**		**0**	100		0	**100**		**0**	100		0	**100**		**0**	100		0
PR	*Providencia rettgeri*	1	0	**100**		**0**	100		0	**100**		**0**	100		0	**100**		**0**	100		0
PR	Aggregated	19	2	**100**	**50**	**1**	100	50	1	**100**	**100**	**2**	95	50	2	**89**	**100**	**4**	79	100	6
PS	*Pseudomonas aeruginosa*	11	0	**91**		**1**	100		0	**100**		**0**	91		1	**91**		**1**	91		1
PS	Other *Pseudomonas*	1	0	**100**		**0**	100		0	**100**		**0**	100		0	**100**		**0**	100		0
PS	Aggregated	12	0	**92**		**1**	100		0	**100**		**0**	92		1	**92**		**1**	92		1
ST	CoNS	17	0	**100**		**0**	94		1	**94**		**1**	94		1	**94**		**1**	82		3
ST	*Staphylococcus aureus*	9	1	**89**	**0**	**1**	100	0	0	**100**	**0**	**0**	100	0	0	**89**	**0**	**1**	56	100	5
ST	*Staph. saprophyticus*	4	0	**100**		**0**	100		0	**100**		**0**	100		0	**100**		**0**	75		1
ST	Aggregated	30	1	**97**	**0**	**1**	97	0	1	**97**	**0**	**1**	97	0	1	**93**	**0**	**2**	73	100	9
EN	*Enterococcus faecalis*	38	4	**87**	**25**	**6**	92	25	4	**87**	**25**	**6**	89	25	5	**76**	**50**	**11**	63	75	17
EN	*Enterococcus faecium*	9	0	**78**		**2**	89		1	**78**		**2**	78		2	**67**		**3**	33		6
EN	Aggregated	47	4	**85**	**25**	**8**	91	25	5	**85**	**25**	**8**	87	25	7	**74**	**50**	**14**	57	75	23
SR	Alpha-hemolytic streptococci	9	0	**78**		**2**	78		2	**78**		**2**	78		2	**78**		**2**	67		3
SR	*Gemella haemolysans*	1	0	**100**		**0**	100		0	**100**		**0**	100		0	**100**		**0**	100		0
SR	*Streptococcus* Group A	1	0	**100**		**0**	100		0	**100**		**0**	100		0	**100**		**0**	0		1
SR	*Streptococcus* Group G	3	0	**100**		**0**	100		0	**100**		**0**	100		0	**67**		**1**	33		2
SR	*Streptococcus* Group C	4	0	**100**		**0**	100		0	**100**		**0**	100		0	**100**		**0**	50		2
SR	Aggregated	18	0	**89**		**2**	89		2	**89**		**2**	89		2	**83**		**3**	56		8
GB	*Streptococcus agalactiae*	18	2	**72**	**50**	**6**	83	50	4	**78**	**50**	**5**	67	50	7	**44**	**50**	**11**	28	50	14
OT	Diphtheroid rod	1	0	**100**		**0**	100		0	**100**		**0**	100		0	**100**		**0**	100		0
OT	*Haemophilus influenzae*	1	0	**100**		**0**	100		0	**100**		**0**	100		0	**100**		**0**	100		0
OT	Aggregated	2	0	**100**		**0**	100		0	**100**		**0**	100		0	**100**		**0**	100		0
NE	Culture negative/no	0	207		**64**	**132**		64	132		**77**	**160**		80	165		**88**	**181**		96	198
MP	Mixed flora G+	0	293		**34**	**100**		36	107		**43**	**127**		45	133		**57**	**166**		78	229
MN	Mixed flora G-	2	200	**100**	**31**	**62**	100	28	55	**100**	**41**	**82**	100	37	73	**100**	**59**	**119**	100	80	160
All	Total	577	735	**94**	**42**	**339**	96	42	330	**94**	**53**	**420**	94	53	421	**90**	**67**	**552**	81	84	732

*SBU* significant bacteriuria, *SE* sensitivity, *SP* specificity, *NC* not cultured (i.e. specimens screened as culture negative), *spp*. species, *CoNS* coagulase negative staphylococci

^a^Group = bacteria or bacterial groups. EC = *E. coli*; KC = *Klebsiella/Citrobacter* group; PR = *Proteus* group; PS = *Pseudomonas* group; ST = Staphylococcal group; SR = Streptococci group; GB = *Streptococcus agalactiae* (GBS); OT = other bacteria; NE = culture negative; MP = Mixed grampositive flora; MN = mixed gramnegative flora; All = total

^b^Methods evaluated: Jolkkonen et al., Manoni et al., De Rosa et al., Flow cell analysis-linear discriminant analysis at 98% sensitivity (FCA-LDA98), FCA-LDA95 = at 95% sensitivity, FCA-LDA90 = at 90% sensitivity

^c^Pos = SBU when urinary tract infection was defined as growth of ≥10^7^ colony forming units per liter at culture

**Table 6:** Outcome of the four screening methods of 1,312 urine specimens examined with flow cytometry analysis when UTI was defined as growth of ≥10^8^ colony forming units per liter in urine at culture

Group^a^	Bacteria	SBU		Methods evaluated^b^																	
				Jolkkonen			Manoni			De Rosa			FCA-LDA_98_			FCA-LDA_95_			FCA-LDA_90_		
		Pos^c^	Neg	SE	SP	NC	SE	SP	NC	SE	SP	NC	SE	SP	NC	SE	SP	NC	SE	SP	NC
EC	*Escherichia coli*	311	82	**100**	**20**	**16**	100	18	15	**100**	**28**	**23**	100	24	20	**100**	**48**	**39**	98	77	70
KC	*Acinetobacter spp*.	0	1		**0**	**0**		0	0		**100**	**1**		0	0		**100**	**1**		100	1
KC	*Citrobacter spp*.	7	2	**100**	**0**	**0**	100	0	0	**100**	**0**	**0**	100	0	0	**100**	**0**	**0**	100	50	1
KC	*Citrobacter freundii*	1	0	**100**		**0**	100		0	**100**		**0**	100		0	**100**		**0**	100		0
KC	*Enterobacter spp*.	2	3	**100**	**33**	**1**	100	33	1	**100**	**33**	**1**	100	33	1	**100**	**33**	**1**	100	33	1
KC	*Other Klebsiella*	1	1	**100**	**100**	**1**	100	100	1	**100**	**100**	**1**	100	100	1	**100**	**100**	**1**	100	100	1
KC	*Klebsiella oxytoca*	9	1	**100**	**0**	**0**	100	0	0	**100**	**0**	**0**	100	0	0	**100**	**0**	**0**	100	100	1
KC	*Klebsiella pneumoniae*	20	13	**95**	**54**	**8**	100	46	6	**100**	**51**	**7**	95	54	8	**95**	**62**	**9**	95	62	9
KC	*Pantoea spp*.	1	0	**100**		**0**	100		0	**100**		**0**	100		0	**100**		**0**	100		0
KC	Aggregated	41	21	**98**	**43**	**10**	100	38	8	**100**	**48**	**10**	98	43	10	**98**	**52**	**12**	98	62	14
PR	*Morganella morganii*	2	3	**100**	**33**	**1**	100	33	1	**100**	**33**	**1**	100	33	1	**100**	**67**	**2**	100	100	3
PR	*Proteus mirabilis*	7	5	**100**	**0**	**0**	100	0	0	**100**	**20**	**1**	86	0	1	**86**	**20**	**2**	86	40	3
PR	*Proteus vulgaris*	3	0	**100**		**0**	100		0	**100**		**0**	100		0	**100**		**0**	100		0
PR	*Providencia rettgeri*	1	0	**100**		**0**	100		0	**100**		**0**	100		0	**100**		**0**	100		0
PR	Aggregated	13	8	**100**	**13**	**1**	100	13	1	**100**	**25**	**2**	92	13	2	**92**	**38**	**4**	92	63	6
PS	*Pseudomonas aeruginosa*	11	0	**91**		**1**	100		0	**100**		**0**	91		1	**91**		**1**	91		1
PS	Other *Pseudomonas spp*.	0	1		**0**	**0**		0	0		**0**	**0**		0	0		**0**	**0**		0	0
PS	Aggregated	11	1	**91**	**0**	**1**	100	0	0	**100**	**0**	**0**	91	0	1	**91**	**0**	**1**	91	0	1
ST	CoNS	10	7	**100**	**0**	**0**	100	14	1	**100**	**14**	**1**	100	14	1	**100**	**14**	**1**	100	43	3
ST	*Staphylococcus aureus*	6	4	**100**	**25**	**1**	100	0	0	**100**	**0**	**0**	100	0	0	**100**	**25**	**1**	83	100	5
ST	*Staphylococcus saprophyticus*	3	1	**100**	**0**	**0**	100	0	0	**100**	**0**	**0**	100	0	0	**100**	**0**	**0**	67	0	1
ST	Aggregated	19	12	**100**	**8**	**1**	100	8	1	**100**	**8**	**1**	100	8	1	**100**	**17**	**2**	89	58	9
EN	*Enterococcus faecalis*	23	19	**96**	**26**	**6**	100	21	4	**100**	**32**	**6**	100	26	5	**91**	**47**	**11**	78	63	17
EN	*Enterococcus faecium*	4	5	**100**	**40**	**2**	100	20	1	**100**	**40**	**2**	100	40	2	**100**	**60**	**3**	75	100	6
EN	Aggregated	27	24	**96**	**29**	**8**	100	21	5	**100**	**33**	**8**	100	29	7	**93**	**50**	**14**	78	71	23
SR	Alpha-hemolytic streptococci	2	7	**100**	**29**	**2**	100	29	2	**100**	**29**	**2**	100	29	2	**100**	**29**	**2**	100	43	3
SR	*Gemella haemolysans*	1	0	**100**		**0**	100		0	**100**		**0**	100		0	**100**		**0**	100		0
SR	*Streptococcus* Group A	0	1		**0**	**0**		0	0		**0**	**0**		0	0		**0**	**0**		100	1
SR	*Streptococcus* Group G	0	3		**0**	**0**		0	0		**0**	**0**		0	0		**33**	**1**		67	2
SR	*Streptococcus* Group C	1	3	**100**	**0**	**0**	100	0	0	**100**	**0**	**0**	100	0	0	**100**	**0**	**0**	0	33	2
SR	Aggregated	4	14	**100**	**14**	**2**	100	14	2	**100**	**14**	**2**	100	14	2	**100**	**21**	**3**	75	50	8
GB	*Streptococcus agalactiae*	6	14	**83**	**36**	**6**	100	29	4	**100**	**36**	**5**	100	50	7	**67**	**64**	**11**	50	79	14
OT	Diphtheroid rod	1	0	**100**		**0**	100		0	**100**		**0**	100		0	**100**		**0**	100		0
OT	*Haemophilus influenzae*	1	0	**100**		**0**	100		0	**100**		**0**	100		0	**100**		**0**	100		0
OT	Aggregated	2	0	**100**		**0**	100		0	**100**		**0**	100		0	**100**		**0**	100		0
NE	Culture negative/no	0	207		**64**	**132**		64	132		**77**	**160**		80	165		**87**	**181**		96	198
MP	Mixed flora Gram+	0	293		**34**	**100**		36	107		**43**	**127**		45	133		**57**	**166**		78	229
MN	Mixed flora Gram-	2	200	**100**	**31**	**62**	100	28	55	**100**	**41**	**82**	100	37	73	**100**	**59**	**119**	100	80	160
All	Total	436	876	**99**	**38**	**339**	100	38	330	**100**	**48**	**420**	99	48	421	**98**	**62**	**552**	95	81	732

*SBU* significant bacteriuria, *SE* sensitivity, *SP* specificity, *NC* not cultured (i.e. specimens screened as culture negative), *spp*. species, *CoNS* coagulase negative staphylococci

^a^Group = bacteria or bacterial groups. EC = *E. coli*; KC = *Klebsiella/Citrobacter* group; PR = *Proteus* group; PS = *Pseudomonas* group; ST = Staphylococcal group; SR = Streptococci group; GB = *Streptococcus agalactiae* (GBS); OT = other bacteria; NE = culture negative; MP = Mixed gram positive flora; MN = mixed gramnegative flora; All = total

^b^Methods evaluated: Jolkkonen et al., Manoni et al., De Rosa et al., Flow cell analysis-linear discriminant analysis at 98% sensitivity (FCA-LDA98), FCA-LDA95 = at 95% sensitivity, FCA-LDA90 = at 90% sensitivity

^c^Pos = SBU when urinary tract infection was defined as growth of ≥10^7^ colony forming units per liter at culture

Also at these screening methods SE had great impact on the proportion of specimens identified as culture negative (26–56%; Tables 2, 3, 5, and 6). For all methods a higher SE was observed for *E. coli* (0–5% above average SE), and lower SE for the enterococci (0 to −18% below average SE) and streptococci groups (0 to −25%) (Tables 2, 5, and 6).

### Prediction of gram groups

Bacteria were predicted to their gram group according to their FCA results such that ln (BC) > −8.49 + 3.82 ln (B-FSC) were classified to be gram-positive otherwise to be gram-negative. In specimens sent to culture, 74–80% of the bacteria were correctly classified to their gram group (Fig. 2).

Calculation of the relative cost in a model including FCA screening found a 5–36% cost reduction compared with non-selective culture depending on the screening method used (Table 3). Implementation of the FCA-LDA_95_ estimated a 22% cost reduction when UTI was defined according to European guidelines (Table 3).

## Discussion

UTIs are a common infection and one of the most commonly analyzed specimens in clinical microbiological laboratories. The aim of the present study was to present and evaluate a new model for identification of culture negative urine specimens identified by FCA.

The basis of the new screening model was to establish the cut off for bacterial and leukocyte counts based on LDA which differs from conventional screening models based on fixed cut off’s for bacteria- and WBC counts (Fig. 1) [11–13, 15–17, 20–22]. The new model was evaluated with respect to: SE, definitions of significant bacteriuria, different uropathogens, group of uropathogens, mixed flora and culture negative specimens and three conventional screening methods [12, 16, 17]. At evaluation the new LDA method proved superior to the Jolkkonen and Manoni methods but similar to that described by De Rosa et al. [17]. The prerequisite for the presented screening method was high SE since diagnosis was later confirmed by urine culture. Since the outcome for SBU defined according to European guidelines was superior compared with 10^7^ CFU/L, irrespective of UTI symptoms. For this reason is the European guidelines recommended.

Currently, the instrument is mainly used for screening to identify and rule out culture negative specimens. However, screening can be done at different levels of SE, e.g. at lower SE for non hospitalized patients as UTIs are often harmless and self eradicating [2] or at higher SE (or omitted) in high risk patients in intensive care, immunosuppressed patients, pregnancy and those with pyelonephritis. In these patients, urine culture is recommended irrespective of the outcome of the FCA screening to avoid missed diagnosis in those deemed high risk. Since *Streptococcus agalactiae* is a potential threat to the unborn child and mother, we always recommend urine culture at pregnancy to identify colonization at low colony counts (<10^6^ CFU/L).

Overall, we found that FCA-LDA_95_ provided a good balance between high SE and specimens excluded as culture negative (> 40%). At our laboratory, nearly 12,600 (of 30,000) specimens could be excluded from culture providing a significant reduction in workload, costs and turnaround time. The predicted cost reduction in the present model (5–36%) is in accordance with others [23]. In addition, FCA analysis adds important information to clinicians in identifying or rejecting patients for antimicrobial treatment. For those treated, the microorganisms Gram group adds further information for treatment success.

The microorganism’s Gram stain identity was predicted by use of B-FSC [24, 25] with an accuracy of 76% (74–80%) in the present study. Rod-shaped bacteria are predicted at higher accuracy than for cocci, >90% and 29%, respectively [26]. Use of NaOH-sodium dodecyl sulfate (SDS) has been proven to facilitate discrimination between Gram positive and Gram negative bacteria [27].

Excluded specimens (urinary catheters and those from pregnant women) were re-examined with a similar outcome as those included (data not shown). LDA screening seems therefore also to be useful for these specimens but further studies are warranted.

*O*f Among the false negative specimens patient 8 had “high” WBC counts and low bacterial counts despite presence of *Proteus mirabilis* at 10^8^ CFU/L at culture. We speculate that the bacterial FCA-staining may have failed in this specimen.

The strength of the present study is the new screening approach, the large cohort and the comprehensive evaluations and against three conventional screening methods [11, 16, 17]. Also the outcomes are presented for different uropathogens, groups of bacteria, negative cultures and pinpoint the strength and weakness in the different methods as the impact of the definition of SBU.

Similar evaluations are, to our knowledge, not reported for SBU defined according to the European definition (at ≥10^6^ CFU/L of an uropathogen with acute uncomplicated cystitis) [7]. In addition we also evaluated the outcome at ≥10^7^ and ≥10^8^ CFU/L irrespective of presence of UTI symptoms.

The distribution of uropathogens was in accordance with other studies [20, 28, 29] and sampling was carried out to minimize the destruction of RBC and WBC [30]. Also, the sampling approach possessed a very low risk for contamination with improved specimen quality.

A weakness is the cohort’s population structure with respect to gender, age, in−/outpatients, type of specimens, etc., which differs from other studies and explains the differences in outcome [11–13, 15–17, 19–22, 31]. However, the pathophysiology of UTI is expected to be quite similar despite some differences between species [9] and cohorts. However, the relatively large differences in outcome between studies is probably due to different cut off’s rather than differences between cohorts, which is supported in the present study where similar outcome was found for the present and De Rosa’s screening model. Also at an optimal cut off we expect a similar outcome, unless one method is superior. If so, a universal cut off can be implicated which significantly enhances implementation of the FCA screening method in clinic, but further studies are warranted.

Also, the delay from initiation until publication is a weakness but the distribution of uropathogens causing UTI is quite conserved over decades [2, 20, 28, 29, 32].

The present cohort may not reflect urine specimens from an average population but rather a selected cohort of specimens from patients with suspected UTI, pyelonephritis and failure or control after treatment. However, the specimens are representative of the urine samples analyzed at our laboratory.

Flow cytometry is a promising method to identify and rule out culture negative urine specimens prior to culture [11–13, 15–17, 19–22, 31]. The present screening model can be recommended in clinic for pre-screening of UTI specimens prior to culture to improve laboratory management of UTI specimens and service to clinicians. By an early identification of culture negative specimens, patients are excluded for unnecessary antimicrobial treatment and its associated side effects.

In summary, a new screening concept is presented based on LDA of FCA data that excludes 42% as culture negative urine specimens prior to culture when UTI was defined according to European guidelines. The present LDA screening method was superior or similar to three conventional methods and is recommended for clinical use to reduce workloads, costs, turnaround time and time to diagnosis.
